# Letter from the St. Louis Editor

**Published:** 1848-01

**Authors:** 


### Dental Register of the West.

VOL. 1.] JANUARY, 1848. [NO. 2.

### LETTER FROM THE ST. LOUIS EDITOR.

### *Editorial Connection with the Register.*

On entering upon the duties assigned us by the "Mississippi Valley Association of Dental Surgeons," it may not be wholly inappropriate on our part to say something in relation to the new field of labor which, as Associate Editor of the "Dental Register of the West," is presented for our examination. We regret that some one more capable and experienced in the duties than ourself had not been selected to aid in advancing what has been so wisely begun by the Association. But a sense of duty to a profession, to the study and practice of which nearly twenty years have been diligently devoted, alone induces us to employ our full abilities in endeavoring to effect something that shall advance its interests and improve its members.

When the present condition of Dental Surgery is contrasted with that which existed a century ago, how wonderful is the change! The mind naturally reverts to that period of its history when it was only practised by men ignorant alike in this as in every other branch of useful knowledge, and whose whole art consisted in the extraction of teeth only, which was performed in a rough and unskillful manner, and frequently causing irreparable injury by the rudely constructed instruments employed in the operation. The opinion was long entertained that a man could easily become a Dentist, no matter what had been his previous occupation or facilities for informing himself in regard to the causes of disease or the remedial agents which should produce their removal. Nor is the latter opinion as yet entirely eradicated; still every member of the Dental profession may congratulate himself that this last relic of stupidity—allied to the barbarous times in which Dentistry was only practised by Barbers—is fast disappearing before the light of intelligence which is now shedding its effulgent rays upon every department of human knowledge, and every subject of human inquiry. Each community expects a Dental Surgeon to be, what undoubtedly he *ought* to be, a prompt and skillful operator, well read in the medical and cognate Sciences, and, upon all occasions, an *accomplished gentleman.*

Is the standard too high? Our professional brethren will unanimously respond *negatively* to the question which implies, in its terms, less *mental* capacity with less mental acquisitions than any, by common consent, allotted to the members of other professions. In short, the standard can never be too high so long as progress marks the present age and leaves its foot prints in the Halls of Science.

In our editorial connection with this Journal it will always be a pleasing duty to treat, with deference and respect, the conscientious opinions of others, however widely they may differ with us, and examine all subjects which may come under our notice candidly, temperately and fearlessly;—to unite all the elements which we may be able to enlist in the interest of the Register, and faithfully contribute our mite to elevate the profession, and diffuse among its members a full knowledge of all recent discoveries which may be deemed useful or necessary in the alleviation of suffering or the repairing of misfortunes. In this noble enterprise, noble in its subjects and in its aims, we can all meet and counsel together.

While in the progress of mutual improvement and interchange of professional sentiment, we shall always feel it to be our duty to treat with uncompromising hostility every *phase* and *shade* of Dental quackery—unfortunately, all professions, and our own more especially, are cursed with empirics—to oppose those men who proclaim to the world their extensive practice, unbounded skill, and peculiar merits above all others, although generally unacquainted with the first principles of Dental Science—men who do not hesitate to undertake the performance of all and every operation known to the profession, which operations, as might reasonably be expected, between *soft solder* and *twelve carat*, are so imperfect that they answer no useful purpose, save to teach the patient, when it is too late, that *cheap* operations and *ignorant* operators are the most costly in the end. Instead, however, of the quack receiving the just obloquy and scorn, so well merited for his ignorance and presumption, too frequently the whole profession suffers in the estimation of the community.

Another form of this hydra-headed monster, *Quackery*, is that which is begotten and nurtured by the aid and influence of hypocrisy. It even pollutes the sanctity of the house of prayer, and prostrates the body before the altar of religion, that it may the better be enabled to accomplish its dark and sinister designs, and to obtain a contemptible subsistence by means too unworthy to be specified. Another form shows itself in Dental cards and flaming advertisements, put forth by men engaged in Dental practice, and endorsed by *Clergymen*, *Generals*, *Colonels*, *Esqs.*, *&c.*, *&c.*, who recommend them for their professional skill. How, in the name of common sense, can either of the above classes judge correctly of the qualifications of a Dental Surgeon? We are happy, however, to bear testimony to the fact that many of the clergy have refused the use of their names and influence to gratify the cupidity of the designing Dental Empiric. Likewise, the too prevalent taste manifested in the display of gaudy instruments, exclusive possession of this or that appliance, and offices covered with imposing signs, as thick as coon skins around a cabin, intimating the wonders contained within, constitute some of the empirical practices of which the profession may justly complain. Other instances might be cited, but we trust these are enough for the present, and will serve the purpose of showing, at least, our detestation of such trickery.

Every Dental Surgeon should be acquainted with Anatomy; not only the Anatomy of the head, but that of the whole body. A full and correct knowledge of every part of the system, as obtained by dissection, is absolutely essential to the proper prosecution of the important duties devolving upon him; for without a proper knowledge of the anatomical structure and relations of all parts of the body, no one is qualified to treat any of the diseases to which the system is so liable. What would be thought of a man who would launch his barque upon the trackless ocean without a chart, compass, or even a knowledge of the starry heavens? No less presumptuous and equally certain of shipwreck is the professed Dental Surgeon who is not thoroughly familiar wlth the science of Anatomy. For example, let us take a case of diseased Antrum, where the patient is suffering all the usual symptoms consequent upon a disturbed condition of the functions of that cavity; he applies to a Dentist who has not considered anatomical knowledge of any benefit to him in his profession; the case is stated, and the examination made; a decayed tooth is discovered under the seat of pain; the patient is informed that the tooth is the cause of the mischief, and extraction is proposed as the remedy, and the operation is performed. If a root, or roots, of the tooth penetrates the Antrum, (as is sometimes the case,) he is astonished on beholding a discharge of matter, which relieves the most urgent symptoms for the time being; if, on the other hand, the roots of the tooth do *not* penetrate the cavity of the Antrum, the extraction affords no relief whatever, and the patient is discharged, with the assurance that the trouble is occasioned by inflammation of the gum, which will pass off in a few days! He continues to suffer a while longer, and again returns to the Dentist for relief; the latter can do nothing more, and refers him to the Physician, who, upon examination, discovers the true state of the case, and adopts measures in accordance therewith. Now, who will not say that the profession will continue to be degraded by such a member, who, from wilful negligence and ignorance of the anatomical structure of the part, is unable to propose paliative or curative measures for the relief of his patient. Cases might be multiplied wherein the advantages of anatomical knowledge is even more *fully* set forth than in the one we have instanced; but, as it is one of many similar instances which have come to our personal knowledge, we prefer giving it as an example.

How can the influence exerted over the general health by a diseased condition of the mouth be explained except by the sympathetic relations, the continuity and contiguity of parts? And how can these terms be understood without an intimate anotomical knowledge of the whole system? And how can the nervous communications existing between the head and stomach be known unless it be derived from a knowledge of Anatomy, obtained by dissection and a close physiological study of the nervous system? Anatomy explains the structure, situation and relations of the various parts or organs of the body; Physiology their functions or uses while in a healthy state: and Pathology relates to the diseased condition of the organs; hence, an imperious duty requires the Dental Surgeon to make himself perfectly familiar in these branches of Medical Science. It is equally important to the successful Dental practitioner that he be acquainted with the practice of medicine, for without it he is unqualified to form a correct diagnosis of the diseases which either may be the cause or be caused by derangements of the organs of mastication; nor is he able to prescribe the appropriate remedies for the removal of such diseases.

A knowledge of Materia Medica will also be found indispensable, as, by means of it, we become acquainted with the history and character of those remedial applications which it is necessary to employ. Chemistry makes known to us the composition of bodies, whether organic or inorganic, and the effects which are produced by the application of various substances to the body, as, for example, acids and alkalies to the teeth, &c., &c. Therapeutics explains the action of medicinal substances upon the system.

Surgery has been defined to be that branch of medicine which principally effects the cure of diseases by the application of the hand alone, the employment of instruments, or the use of topical remedies. Natural History and Comparative Anatomy offer equal inducements for their study by the opportunities which they present for the acquisition of a knowledge of the various phases of dentition. A knowledge of Metallurgy will materially facilitate the labor of the Dental Surgeon, and, in short, that a thorough medical and scientific knowledge is absolutely necessary to any man who now practices the profession is evident, from the fact that cases which were formerly supposed to belong to the Physician or Surgeon alone, now properly come under the care of the Dental Surgeon.

Although the tone of the Dental profession has undergone a complete revolution in the last few years, yet we must not forget that this is mainly attributable to the timely and long continued exertions of certain distinguished members of the profession, who have truly rendered themselves deserving of every honor for the many and great sacrifices which they have been forced to make in order to effect this most noble and meritorious object.

Every person, therefore, who aspires to enter upon the duties pertaining to the Dental profession should be substantially versed in the medical and cognate sciences, with a critical knowledge of the mechanism necessary to success in the Dental Laboratory; hence, the hands as well as the head must be educated, and the power of applying the prescribed combinations with readiness must be acquired. Habits of close observation, together with untiring perseverance, can alone obtain for the aspirant that point of professional excellence, to aim at reaching which is the indispensable duty of every one now engaged in or about to enter upon the profession.

A cause productive of great evil may be found in the members of the profession attempting to qualify young men for the duties of practice in too short a period. They are received for a certain fee, and, generally, the *fee* is the only inducement, with the condition to remain a length of time varying from three months to a year, in which short space they are considered as having acquired *all* the *knowledge* and *accomplishments* known to their preceptors, and therefore sent forth into the world to perform the important offices which require the most mature intelligence and experience. This condition of things may be remedied by prolonging the term of study to Five years; the first three of which should be devoted to the Laboratory and Operating Room, text books and examinations; and the two last to Lectures, Miscellaneous reading and practice. We are of the opinion that this is barely sufficient to qualify a Dental Student for the duties of his profession. True, it is considered by many well informed persons too great a length of time to be applied to the study; but our experience has proven it to be absolutely essential to this proper qualification of a Dental Surgeon. During this probational period, the student will, to a great extent, acquire that *tact* in the use of the instruments and tools which is demanded in the various manipulations attending the medical, surgical and mechanical departments.

Happily the Dental profession possesses the means within itself by which the evil complained of may, in time, be somewhat mitigated, if not entirely removed; thus leaving no excuse for any one who fails to avail himself of the advantages which are now liberally placed before him. I refer to the Colleges of Dental Surgery at Baltimore and Cincinnati, now successfully established and conducted by Professors of acknowledged talent and merit, where a thorough and scientific course of study is diligently enjoined and faithfully pursued.—ST. L. ED.

